# *In silico* prediction of metabolic trait robustness in microbial cells

**DOI:** 10.1371/journal.pcbi.1014864

**Published:** 2026-10-27

**Authors:** Chunhao Gu, Ville Mustonen, Paula Jouhten

**Affiliations:** 1 Department of Bioproducts and Biosystems, Aalto University, Espoo, Finland; 2 Department of Computer Science, Department of Organismal and Evolutionary Biology, University of Helsinki, Helsinki, Finland; Universita degli Studi di Milano-Bicocca, ITALY

## Abstract

Microbial strains undergo notable biomass expansion in industrial applications subjecting populations to Darwinian selection. The consequent adaptive evolution may threaten the often tediously developed desired metabolic traits of the strains, such as flavor or platform chemical production. Yet, it remains unresolved how to predict the evolutionary trait robustness. Here, we propose TRAEV (Trait Robustness Against EVolution), a computational framework for *in silico* prediction of evolutionary robustness of metabolic traits and trajectories of desired traits in application environments. TRAEV uses constraint-based metabolic models for predicting environment-dependent trait-fitness dependencies and accounts for the stochasticity of adaptive evolution by using Monte Carlo-based perturbation analysis. First, TRAEV predicts the immediate metabolic phenotype in the new environment, and then, the subsequent evolutionary trajectories of fitness and desired trait. Metabolic trait robustness is quantified from the evolutionary trajectories as two scores: Robustness Score (RS) and Trade-off Score (TS). RS represents trait robustness and the overall trait level during evolutionary adaptation, whereas TS captures trait–fitness trade-offs. We validated TRAEV by predicting relative evolutionary robustness of heterologous pigmentation of two engineered *Saccharomyces cerevisiae* strains and two desired aroma generation traits by multiple wine strains of *S. cerevisiae* if they were developed via a laboratory selection process. Thus, we showed how TRAEV predictions could guide strain development. TRAEV can be integrated into computational strain design workflows across microbial strains, desired metabolic traits, and application environments. Ultimately, model-predicted evolutionary robustness of desired metabolic traits can guide both strain and process development and help enhancing the economic attractiveness of industrial applications using microbial cells.

## Author summary

Microbial cells developed to express desired traits by genetic engineering or selection processes are used in a wide range of applications, from biotechnological chemical production to food and beverage fermentations. However, when the cells replicate in the application environment the traits are subject to Darwinian selection and consequent adaptive evolution. As a result, the desired traits can be compromised. To *in silico* predict the evolutionary adaptation of desired traits we developed TRAEV (Trait Robustness Against EVolution), a computational framework for assessing evolutionary trajectories and robustness of desired metabolic traits in application environments. TRAEV integrates constraint-based modeling and Monte Carlo-based perturbation analysis. We validated and demonstrated the usability of TRAEV by predicting the evolutionary robustness of heterologous pigmentation of two engineered *Saccharomyces cerevisiae* strains, and aroma generation by multiple *S. cerevisiae* wine strains if they were developed by laboratory selection. Usable across microbial strains, metabolic traits, and application environments, we believe that TRAEV can contribute to the development of economically attractive industrial applications using robust traits expressing microbial cells.

## Introduction

In biotechnology, microbial strains are developed by genetic engineering [1,2] or through artificial or natural selection [3,4] to enhance the desired traits, most notably, overproduction of target compounds. Such engineered or selected microbial strains can be used for industrial production of fuels, platform chemicals [5], drugs [6], and aromas, flavors, and preservatives in food and beverages [7]. However, the enhanced desired traits inevitably divert translational capacity and/or metabolic precursors away from processes associated with cell growth [8,9]. This reallocation commonly compromises cellular fitness. Thereby, the risk of evolutionary non-robustness of desired trait is elevated and a lessening or loss of the initially enhanced trait may occur over time [10].

Previously, the focus has been on achieving optimal enhancement of desired traits [11–13], but the evolutionary robustness of these traits in industrial application environments has been overlooked. However, the enhanced engineered or selected traits that allow microbial cells to achieve the desired application performance are subject to Darwinian evolution because of their prevalent fitness effects (i.e., the negative covariance between desired traits and cellular fitness) [14]. Consequently, desired trait robustness, i.e., the ability to maintain the trait under environmental (e.g., nutrient availability, pH) and genetic (e.g., mutations) perturbations and across multiple evolutionary generations, remains a fundamental challenge for large-scale industrial applications [10]. Attempts to develop strains with higher desired trait robustness have been made by coupling traits to cell growth, either through the use of product sensors [15] or by appropriately reducing the metabolic network [16,17]. Nevertheless, the cells have been found to sometimes escape the engineered coupling [18] and sensor systems’ operational robustness in evolutionary time-scales is to be assessed. Therefore, there is a pressing need for a widely applicable approach to predict evolutionary trait trajectories of developed strains, which are pivotal for attractive biotechnology processes [19,20].

Over the past decade, computational modeling, particularly constraint-based modeling, has become an indispensable tool for rational strain design [21]. Genome-scale metabolic models (GEMs) are *in silico* reconstructions of cellular metabolism, including metabolites, reactions, and gene–protein–reaction associations [22]. Constraint-based modeling that combines GEMs and analysis techniques such as flux balance analysis (FBA) [23] and minimization of metabolic adjustment (MOMA) [24] has demonstrated versatile applications in strain development, including simulating flux distributions [23], identifying desired trait enhancing genetic engineering targets [25], predicting metabolic bottlenecks [26], and designing selection environments [27].

Here, we developed a novel computational framework, TRAEV (Trait Robustness Against EVolution), which integrated constraint-based modeling and Monte Carlo-based perturbation analysis to predict relative evolutionary robustness of metabolic traits across strains and application environments. TRAEV takes advantage of GEMs allowing to infer chemical environment-dependent metabolic trait-fitness couplings from first principles [27]. It uses constraint-based GEM simulations to predict engineered or laboratory selected strain’s immediate metabolic trait state in an application environment due to phenotypic plasticity and the evolutionarily adapted state. Based on these states, TRAEV estimates evolutionary trajectories using a Monte Carlo-based perturbation analysis method, from which the desired trait’s robustness is assessed and quantified as the Robustness Score (RS) and the Trade-off Score (TS). We demonstrated TRAEV for predicting the evolutionary robustness of heterologous pigmentation of engineered *Saccharomyces cerevisiae* strains and for *in silico* screening of adaptive laboratory evolution (ALE) environments to develop *S. cerevisiae* strains exhibiting robust aroma generation in wine fermentation.

## Results

### TRAEV for predicting trait robustness in evolving cells

Microbial strains expressing enhanced desired traits are typically developed through genetic engineering (GE), or selection such as ALE, or a combination of both, under controlled laboratory conditions. However, when these strains are taken to industrial applications they become introduced into a new chemical environment (e.g., wine must or corn stalk hydrolysate media), eliciting immediate metabolic adjustments due to phenotypic plasticity [28,29]. This is subsequently followed over time by genotypic changes in the population (i.e., evolutionary adaptation) driven by Darwinian selection (Fig 1A). Both processes can alter the expression of desired traits. Therefore, an approach for predicting trait evolutionary robustness in application environments is called for.

**Fig 1:**
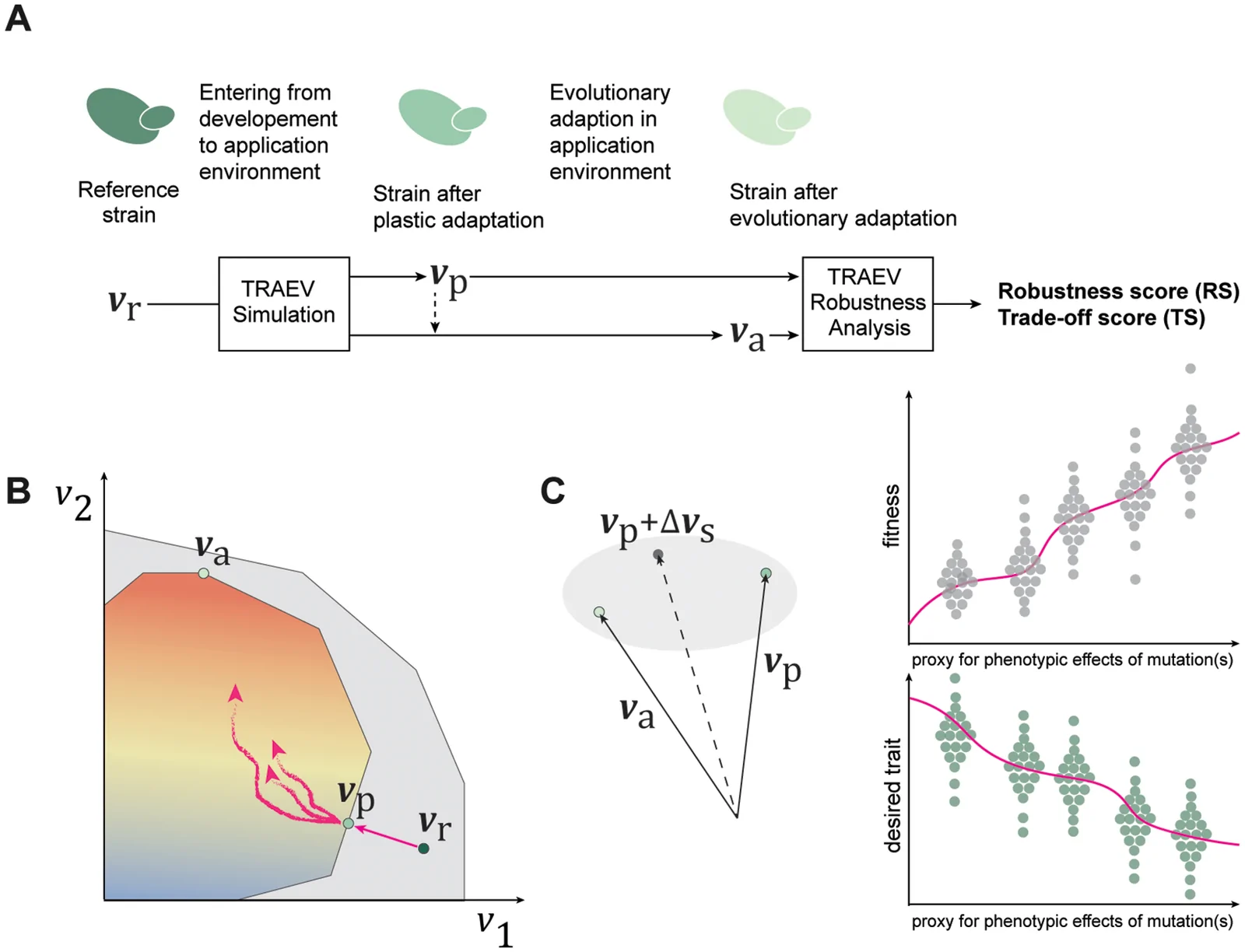
Schematic overview of TRAEV. **(A)** Workflow of TRAEV. The reference state (**v**_r_) represents a metabolic trait (i.e., a set of metabolic fluxes and/or enzyme usages) state of a strain developed, e.g., through genetic engineering (GE) or adaptive laboratory evolution (ALE) in a development environment (i.e., laboratory conditions) and computed using pFBA. With **v**_r_, TRAEV Simulation calculates the strain’s immediate phenotypically adjusted state (**v**_p_) and its evolutionarily adapted state (**v**_a_) in an application environment. Based on these states, TRAEV Robustness Analysis estimates evolutionary trajectories and corresponding Robustness Score (RS) and Trade-off Score (TS). **(B)** Conceptual rationale of TRAEV Simulation. A two-dimensional flux space is defined by fluxes ($v_{1},v_{2}$). Within this space, the gray polygon represents the feasible space in the development environment, within which the strain achieves the reference state **v**_r_ before being introduced into the application environment. The colored polygon represents the feasible space in the application environment, within which **v**_p_ is the state nearest to **v**_r_, while **v**_a_ is the fitness-optimal state where adaptive evolution via Darwinian selection would on average move the cells to. **(C)** Illustration of TRAEV Robustness Analysis. On the left, an evolutionarily intermediate state is represented as ${𝐯}_{p}+\Delta {𝐯}_{s}$, where $\Delta {𝐯}_{s}={𝐦}_{s}⊙({𝐯}_{a}-{𝐯}_{p})$ and ${𝐦}_{s}$ is a binary mask indicating a randomly selected subset ***s*.** On the right, TRAEV characterization of evolutionary trajectories by deriving the fitness and desired trait of intermediate states along progressive changes in enzyme usage (i.e., proxies for phenotypic effects of mutations) for assessing metabolic trait evolutionary robustness is visualized.

To this end, we developed TRAEV, a computational framework for predicting trait robustness in cells adaptively evolving in application environments. TRAEV employs constraint-based GEM simulations to predict both phenotypic plasticity and evolutionary adaptation. Phenotypic plasticity and evolutionary adaptation are not fully time-separated but after immediate plastic response to exposure to new chemical environment the evolutionary adaptation invokes subsequent plastic adaptations to genotypic changes due to mutations. TRAEV uses GEM simulation algorithms minimization of metabolic adjustment (MOMA) [24,30] and regulatory on/off minimization (ROOM) [31] for predicting plastic adaptation and evolutionary adaptation, respectively. MOMA is likely to distribute the adaptive change more widely in the metabolic network while ROOM minimizes the number of adaptive changes to a reference. Thus, TRAEV uses MOMA simulations for predicting plastic adaptation similarly to how it has previously been applied in [29] and ROOM simulations for predicting likely observed evolutionary adaptations via a minimal number of beneficial mutations. Finally, TRAEV Simulation results are analyzed to derive evolutionary trajectories and to assess desired trait’s evolutionary robustness. Specifically, TRAEV consists of two main modules: Simulation and Robustness Analysis.

As shown in Fig 1A, the Simulation takes as input a reference state (**v**_r_), which represents the metabolic state of a developed strain (e.g., an engineered or laboratory-evolved strain). In this study, a metabolic state is defined as a vector containing both metabolic fluxes and corresponding enzyme usages (see Methods), a metabolic trait as a set of metabolic fluxes and/or enzyme usages [27,32] and biomass yield (i.e., growth rate normalized by the total uptake of carbon and nitrogen sources from the chemical environment) was defined as a proxy of Darwinian fitness. **v**_r_ can be computed using parsimonious flux balance analysis (pFBA) [33]. Then, the Simulation calculates two key metabolic states in the application environment:

1. **v**_p_: the immediate metabolic state due to phenotypic plasticity after switching to the application environment, calculated using linear MOMA. This state minimizes the distance from **v**_r_ while ensuring a biologically viable minimal growth rate.
2. **v**_a_: the adapted metabolic state after evolution in the application environment, calculated using the ROOM-based iterative search algorithm (Algorithm 1). This state achieves optimal fitness with fewest changes in enzyme usage (i.e., phenotypic effects of mutations) relative to **v**_p_.

Enzyme usage variables represent the total flux of reactions catalyzed by the enzyme [32]. Thus, enzyme usage change mimics a change in the activity of a single enzyme potentially due to genetic mutations. ROOM algorithm allows minimizing the number of enzyme usage variables whose values have to change with respect to a reference, here **v**_p_. Therefore, ROOM solution represents a proxy of metabolic phenotype response to least mutations. A schematic illustration of the rationale of the Simulation is shown in Fig 1B.

In Robustness Analysis, TRAEV employs a Monte Carlo-based perturbation analysis to predict an ensemble of evolutionary trajectories comprised of intermediate metabolic states between **v**_p_ and **v**_a_. As illustrated in Fig 1C, the resulting evolutionary trajectories are characterized as cells’ fitness and desired trait expression along progressive changes in enzyme usages during adaptive evolution. Finally, two scores are derived to quantify the trait robustness in the application environment:

1. Robustness Score (RS): a metric defined as the mean, over evolutionarily intermediate metabolic states, of the normalized desired trait (Eq. 3), reflecting the evolutionary robustness and overall level of the desired trait. (as defined in Eq. 8).
2. Trade-off Score (TS): a metric defined as the Pearson correlation coefficient, over evolutionarily intermediate metabolic states, between the changes (relative to **v**_p_) in the desired trait and in fitness, representing the trait-fitness trade-off (i.e., negative coupling). (as defined in Eq. 9).

The Robustness Score (RS) ranges from 0 to 1, with higher values indicating higher evolutionary robustness and/or overall level of the desired trait against adaptive perturbations in metabolism. When comparing the evolutionary robustness of the same desired trait across multiple strains, RS is calculated by normalizing the trait with the maximum trait expression among the strains, thereby representing not only evolutionary robustness but also trajectory-averaged attainable level of the desired trait. In contrast, the Trade-off Score (TS) ranges from -1–1, with values less negative indicating weaker trade-off between the desired trait and fitness, thereby suggesting long-term evolutionary robustness of the trait. Together, RS and TS provide a quantitative framework for predictive assessment of strain performance and evolutionary robustness in application environments suited for guiding rational strain design for industrial applications.

The implementation details of TRAEV are provided in the Methods section and the symbols and abbreviations are summarized in Table D in S1 Text.

### Predicting evolutionary robustness of engineered pigment production

Biosyntheses of blue indigoidine and red bikaverin are natural traits of certain bacteria (e.g., *Streptomyces* spp.) and fungi (e.g., *Fusarium* spp.), respectively. However, natural pigment synthesis is subject to complex regulation and is typically not induced under standard laboratory or industrial cultivation conditions. To overcome this limitation, heterologous production of indigoidine and bikaverin has been successfully achieved in *S. cerevisiae*, a common biotechnology host, by introducing their biosynthetic pathways via genetic engineering [11,12].

Here, we revisited the results from a recent experiment [34], where *S. cerevisiae* CEN.PK113-7D and S288C strains were engineered for the production of indigoidine and bikaverin, respectively. ALE of the engineered strains was performed in two distinct media: a chemically defined medium containing ammonium as the sole nitrogen source and D-galactose as the sole carbon source (YNBG), and a rich medium containing peptone as the nitrogen source and D-galactose as the sole carbon source (YPG). In both media, after 25 transfers (i.e., ca. 175 generations in YNBG and ca. 200 generations in YPG), the bikaverin-producing colonies remained mostly pigmented, while the indigoidine-producing lineages had lost nearly all production. These results indicated that the bikaverin-producing strain exhibited higher desired trait evolutionary robustness during prolonged cultivation (Fig 2A and 2B).

**Fig 2:**
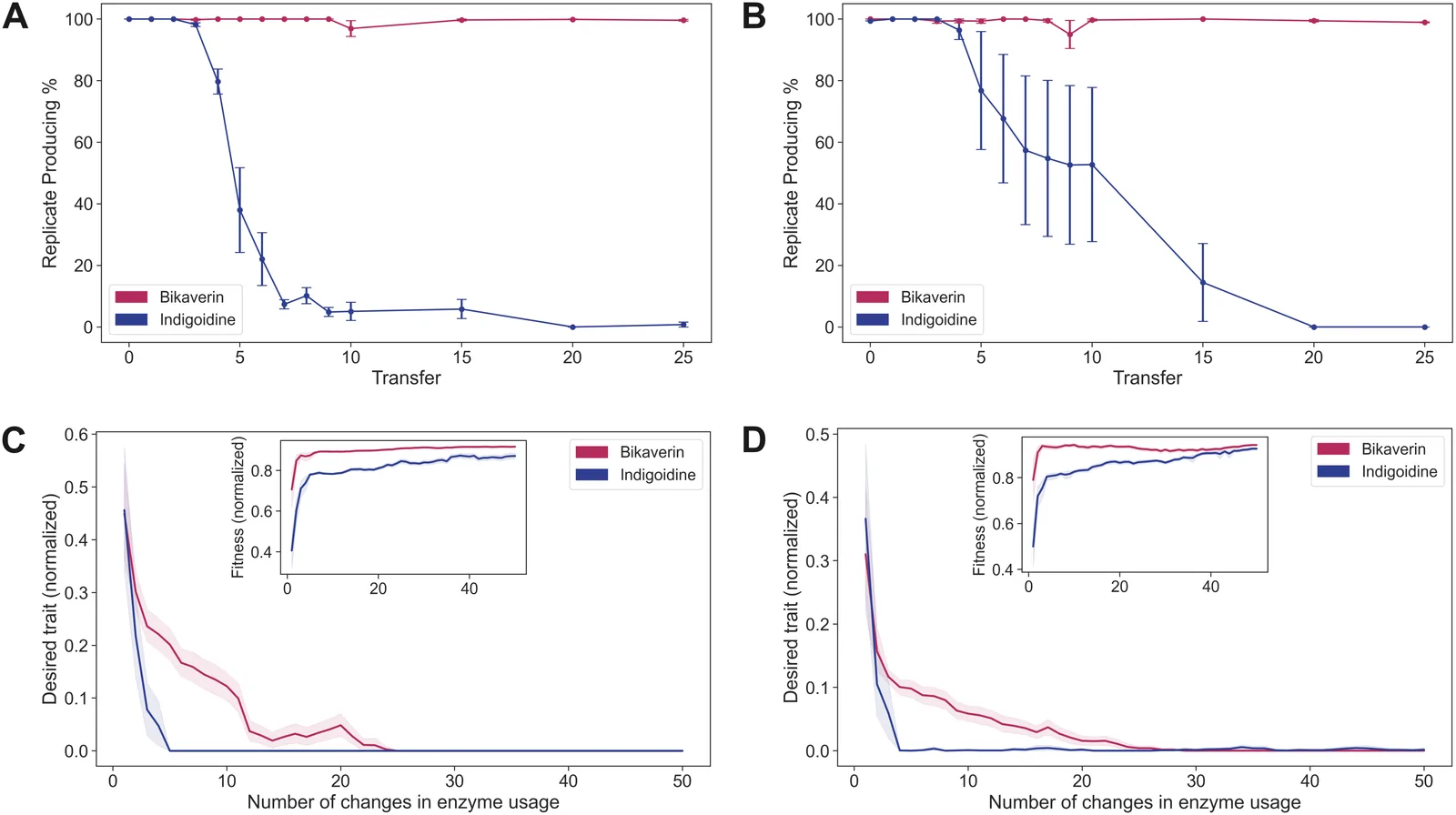
Experimental and TRAEV-predicted evolutionary trajectories of pigment-producing *S. cerevisiae* strains. **(A, B)** Experimental trait expression trajectories showing the percentage of pigmented colonies over 25 transfers in YNBG (A) and YPG (B) media, of bikaverin-producing strain (red) and indigoidine-producing strain (blue), based on data from [34]. Each point with an error bar represents the mean (± SD) of four biological replicates. **(C, D)** TRAEV-predicted trait expression trajectories illustrating changes in normalized desired trait (i.e., pigment excretion flux) vs. the number of changes in enzyme usage from 1 to 100 in YNBG (C) and YPG (D) media of bikaverin-producing strain (red) and indigiodine-producing strain (blue). Each point represents the mean of sampled metabolic states; shaded areas denote 95% confidence intervals. Insets show normalized fitness vs. number of enzyme usage changes.

To validate whether these observations could be computationally predicted, we simulated the experiments using pFBA and TRAEV. The *S. cerevisiae* GEM [35] was augmented with the heterologous pathways for producing indigoidine [11] and bikaverin [12], respectively. pFBA was used to calculate the reference metabolic state **v**_r_ for each engineered strain. With **v**_r_ as the reference state of each strain, TRAEV was then used to predict ALE in YNBG and YPG environments, yielding the immediate phenotypically adapted metabolic state (**v**_p_) and the evolutionarily adapted metabolic state (**v**_a_). Subsequently, Robustness Analysis was applied to predict the evolutionary trajectories and calculate the corresponding RSs (Fig 2C and 2D). Implementation details are provided in the Methods section.

As shown in Fig 2C and 2D, the bikaverin-producing strains exhibited a more gradual decline in pigment production than the indigoidine-producing strains, indicating higher evolutionary robustness of the trait. These simulated evolutionary trajectories agreed qualitatively with the experimental observations (Fig 2A and 2B). In the YNBG environment, the bikaverin-producing strain had RS of $4.75\times 10^{-2}$, compared to $1.32\times 10^{-2}$ for the indigoidine-producing strain. In the YPG environment, the difference in RS was also pronounced, with values of $2.84\times 10^{-2}$ for the bikaverin-producing strain and $9.57\times 10^{-3}$ for the indigoidine-producing strain, respectively. Together, these results showed that TRAEV could be used to predict and rank desired trait evolutionary robustness of strains in application environments before experimental testing. Sensitivity analyses yielded similar conclusions (Section 2 in S1 Text and S1 Fig), suggesting that the findings were stable across different parameter settings.

### Model-guided design of ALE environments for adaptively evolving robust metabolic traits

In a previous study [27], a constraint-based modeling method named EvolveX was introduced to identify selection environments for ALE that promote strains to evolve enhanced level of a desired trait once transferred to the application environment. This strategy relies on a tacking trait, a metabolic trait that is coupled to fitness in the selection environment and to the desired trait in the application environment. However, in the application environment, neither the tacking trait nor the desired trait is necessarily coupled to fitness. Consequently, following evolutionary adaptation driven by Darwinian selection, trait evolutionary robustness in the application environment remains uncertain based on the EvolveX scores.

Here, we used TRAEV to re-evaluate the scoring of selection environments for ALE reported in [27], aiming to obtain robust production, in wine must environment, of two classes of aroma compounds: (1) phenylethyl alcohol and its acetate ester (PEA) and (2) branched-chain amino acid-derived higher alcohols and their esters (BCHA). We designed 1,171 distinct selection environments (see S2 Data), each comprising three nutrients as carbon and nitrogen sources. For each selection environment, we used *S. cerevisiae* GEM [35] and pFBA with nutrient and growth rate constraints to calculate the adapted metabolic state (**v**_r_) in that selection environment. With **v**_r_ as the reference state of each strain, TRAEV was then performed to predict adaptive evolution in anaerobic wine must-like conditions with D-glucose, D-fructose, and yeast assimilable nitrogen (ammonium and free amino acids) as the carbon and nitrogen sources, obtaining the immediate phenotypically adapted state (**v**_p_) and the evolutionarily adapted metabolic state (**v**_a_) for each strain. Finally, for both classes of aroma compounds, Robustness Analysis was conducted to predict the evolutionary trajectories along changes in enzyme usage and calculate the corresponding RS and TS. Implementation details are provided in the Methods section.

In TRAEV Simulation results, strains evolved in 322 of the 1171 selection environments were capable of producing PEA in the application environment, whereas the strains evolved in 499 of these selection environments were capable of producing BCHA. These cases yielded valid Robustness Analysis results for deriving evolutionary trajectories. In Fig 3, we plotted the predicted evolutionary trajectories of PEA production trait in ten ALE developed strains (Fig 3A and 3C) and BCHA production trait in ten ALE developed strains (Fig 3B and 3D). The corresponding RS and TS/ (Eqs. 8, 9) are summarized in Table 1. These examples were randomly chosen from the ALE developed strains ranked in the top 50% by RS, making the trajectories representative of the evolutionary robustness profiles for each trait.

**Table 1:** RS and TS of ALE developed *S. cerevisiae* strains in the application environment.

PEA production			BCHA production		
Selection env.	RS	TS	Selection env.	RS	TS
EtOH,Gal,Phe	$6.21\times 10^{-2}$	$-2.87\times 10^{-1}$	Ala,Arg,Ser	$8.35\times 10^{-2}$	$-7.74\times 10^{-2}$
EtOH,Glc,Phe	$5.43\times 10^{-2}$	$-1.09\times 10^{-1}$	EtOH,Arg,Glu	$9.85\times 10^{-2}$	$1.62\times 10^{-2}$
Gal,Arg,NH_4_	$3.89\times 10^{-2}$	$5.76\times 10^{-2}$	Gal,Ile,Met	$1.23\times 10^{-1}$	$-4.75\times 10^{-1}$
Gal,Asn,Glu	$4.69\times 10^{-2}$	$1.35\times 10^{-1}$	Gal,Ile,NH_4_	$1.12\times 10^{-1}$	$-3.34\times 10^{-1}$
Gal,Glu,Trp	$4.41\times 10^{-2}$	$3.14\times 10^{-1}$	Glc,Gol,NH_4_	$7.50\times 10^{-2}$	$-1.63\times 10^{-1}$
Gal,Gol,Ser	$3.28\times 10^{-2}$	$-1.42\times 10^{-2}$	Glc,Leu,NH_4_	$1.33\times 10^{-1}$	$-2.64\times 10^{-1}$
Glc,Ala,Lys	$5.28\times 10^{-2}$	$3.24\times 10^{-1}$	Glc,Leu,Val	$1.45\times 10^{-1}$	$-2.83\times 10^{-1}$
Glc,Ile,Thr	$3.60\times 10^{-2}$	$1.56\times 10^{-1}$	Glc,NH_4_ ,Ser	$8.71\times 10^{-2}$	$1.79\times 10^{-2}$
Gol,Lys,Phe	$5.62\times 10^{-2}$	$-5.75\times 10^{-1}$	Gol,Gly,Lys	$1.05\times 10^{-1}$	$7.38\times 10^{-2}$
Gol,Met,Phe	$5.90\times 10^{-2}$	$-3.12\times 10^{-1}$	Gol,Ile,Lys	$1.31\times 10^{-1}$	$-3.24\times 10^{-1}$

Gal: D-galactose, Glc: D-glucose, Gol: glycerol, EtOH: ethanol, NH_4_: ammonium, and standard three-letter symbols of amino acids.

**Fig 3:**
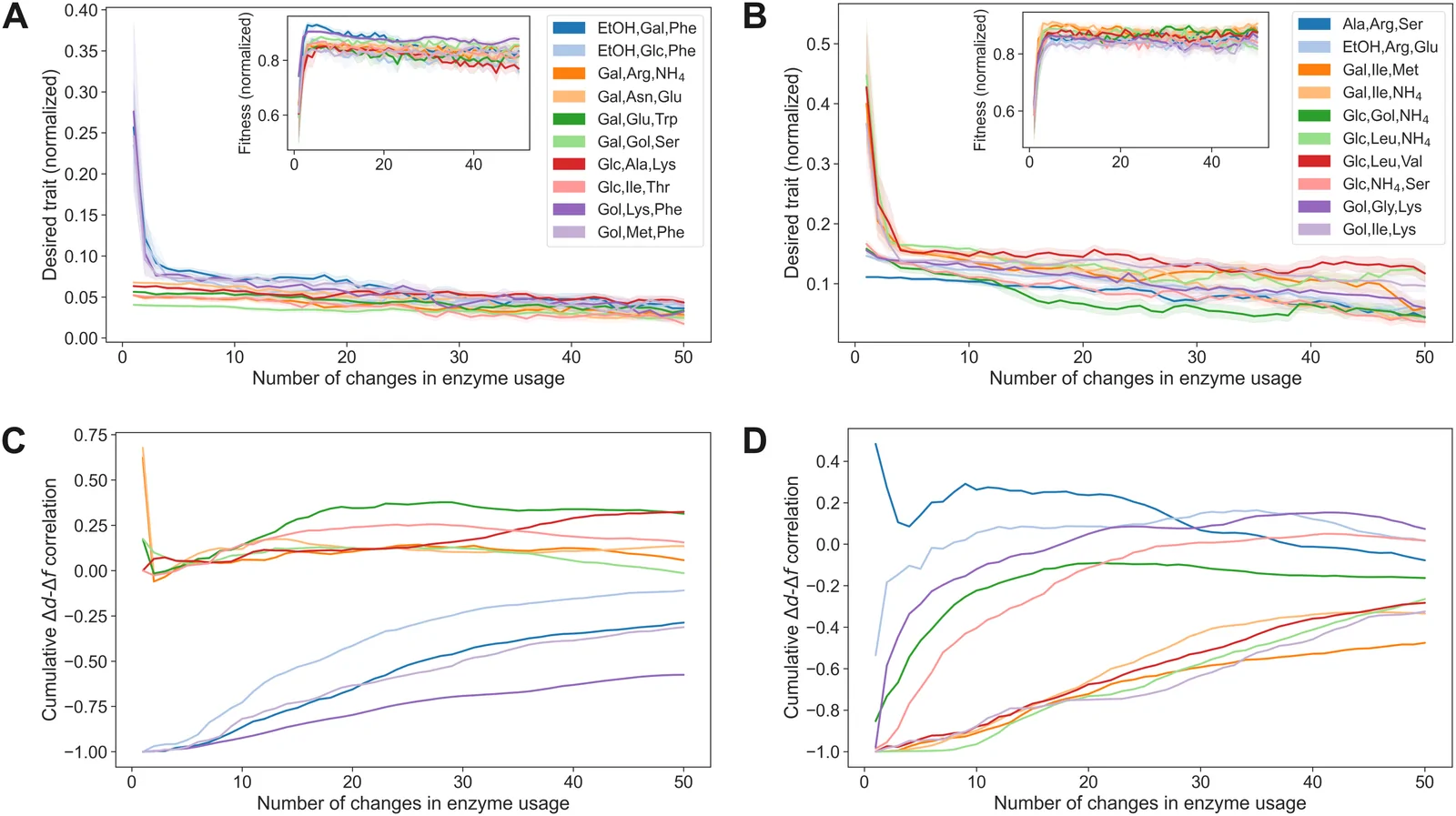
Evolutionary trajectories of ALE developed *S. cerevisiae* strains in the application environment. Evolutionary trajectories are shown as a function of the number of changes in enzyme usage (k=1~50) for strains evolved in example selection environments for enhancing the PEA and BCHA production traits, respectively. **(A, B)** Normalized PEA production trait (A) and BCHA production trait (B) as a function of ***k*.** Each point represents the mean of up to 100 sampled trajectories (except for *k* = 1, where all feasible samples are included), and shaded regions indicate 95% confidence intervals. Insets show the corresponding normalized fitness trajectories. Colors denote different trajectories, and legend entries indicate the nutrient compositions of the corresponding selection environments. **(C, D)** Cumulative correlation between changes in the PEA production trait (C) and BCHA production trait **(D)** (Δd) and changes in fitness (Δf) along the trajectories as a function of ***k*.** Increasingly negative values indicate stronger trade-offs between the desired trait and fitness.

Both PEA and BCHA production traits declined with increasing number of changes in enzyme usage as fitness improved in the application environment (Fig 3A and 3B), accompanied by changing trait-fitness trade-off (Fig 3C and 3D). For the PEA production trait, the strains evolved in selection environments containing the direct precursor of the desired aromas, L-phenylalanine, (e.g., EtOH,Gal,Phe; EtOH,Glc,Phe; Gol,Lys,Phe; Gol,Met,Phe) maintained relatively higher trait levels throughout the trajectories (Fig 3A), resulting in markedly higher RS (Table 1). However, these strains also exhibited among the lowest (i.e., most negative) TS (Fig 3C, Table 1), indicating stronger trade-offs with fitness. Similarly, for the BCHA production trait, the strains evolved in selection environments containing branched-chain amino acids (e.g., Gal,Ile,NH_4_; Glc,Leu,NH_4_;Glc,Leu,Val; Gol,Ile,Lys) tended to retain higher trait levels (Fig 3B) and thus higher RS (Table 1), but at the expense of more negative TS (Fig 3D, Table 1).

Overall, the rankings of strains based on RS and TS were largely opposite across examples, highlighting a tension between maintaining trait robustness and weakening its coupling to fitness. Among the PEA production examples, the strains evolved in selection environments not containing L-phenylalanine showed a relatively low RS but higher TS (Fig 3A and 3C, Table 1). Likewise, for the BCHA production examples, Ala,Arg,Ser and EtOH,Arg,Glu had markedly lower RS but higher TS (Fig 3B and 3D, Table 1). These cases of higher TS indicated a weaker negative coupling between the desired trait and fitness, making such strains promising candidates for further development to achieve higher evolutionary robustness.

To identify groups of selection environments with similar characteristics, we performed K-means clustering analysis using RS and TS calculated for each environment. As shown in Fig 4A and 4B, the selection environments were partitioned into four clusters for both aroma production traits. For the PEA production trait, Cluster 3 was characterized by high RS and markedly low TS relative to the other clusters, indicating environments associated with higher PEA production but stronger trade-offs between the trait and fitness. For the BCHA production trait, Cluster 3 exhibited a similar pattern of high RS and strongly negative TS, whereas Cluster 1 occupied an intermediate region with relatively balanced RS and TS values. Sensitivity analyses based on these cluster assignments yielded similar conclusions (Section 2 in S1 Text and S2 Fig), suggesting that the observed patterns were stable across different parameter settings.

**Fig 4:**
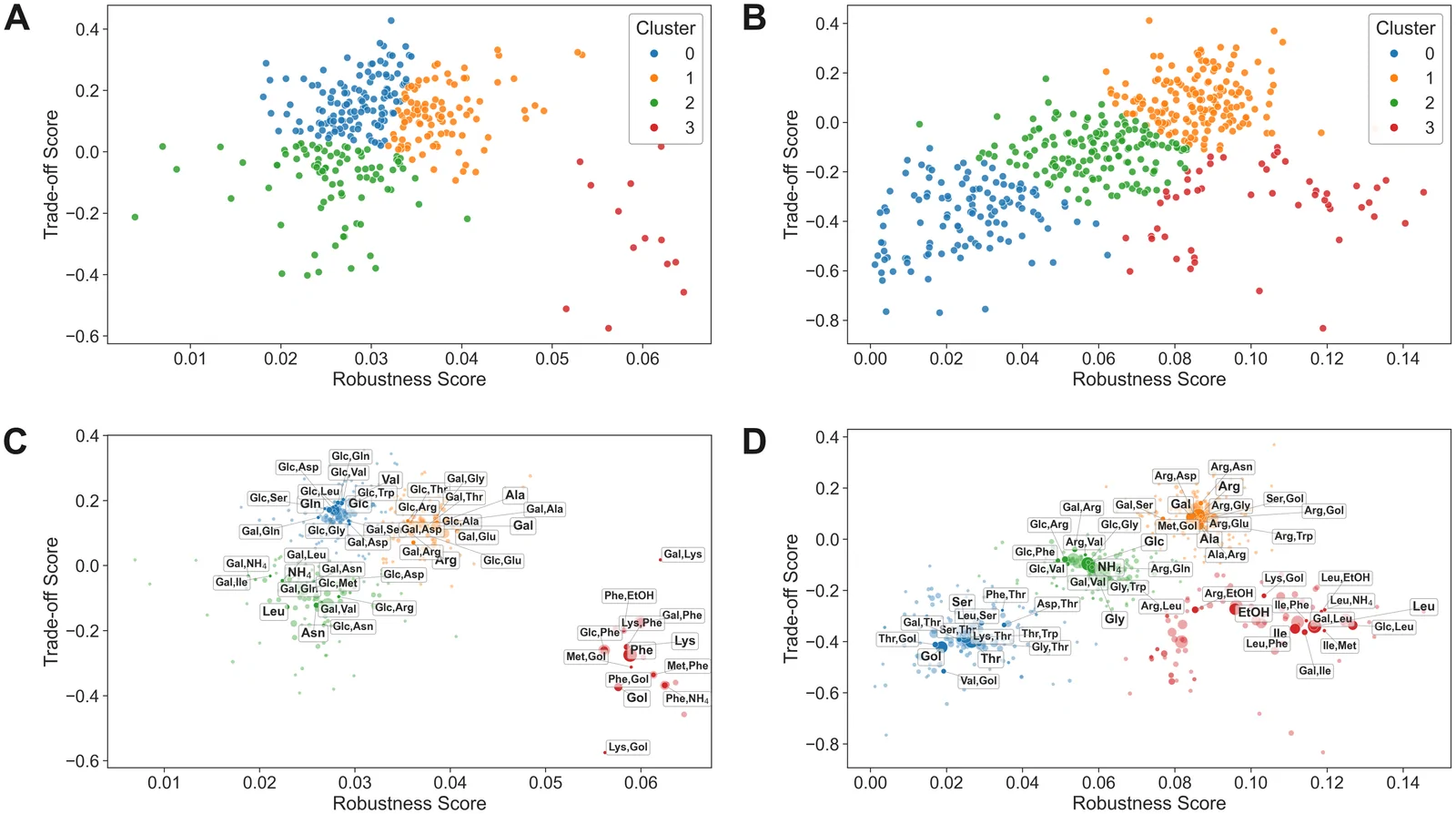
Selection environment clustering and nutrient enrichment. **(A, B)** K-means clustering of selection environments based on RS and TS. Each dot represents a selection environment and colors indicate cluster assignments for PEA production trait (A) and BCHA production trait **(B)**. **(C, D)** Nutrient enrichment analysis for the identified clusters for PEA production trait (C) and BCHA production trait **(D)**. Each dot represents an enriched nutrient or nutrient pair, colored according to the corresponding cluster. Dot size is proportional to the number of selection environments containing the nutrient or nutrient pair. Nutrients with the top three Enrichment Scores (ES) and nutrient pairs with the top ten ES values in each cluster are annotated.

To further investigate the nutrient features of these clusters, we performed nutrient enrichment analysis for both single nutrients and nutrient pairs. As shown in Fig 4C and 4D, for the PEA production trait, Cluster 3 was strongly enriched for L-phenylalanine and glycerol. For the BCHA production trait, Cluster 3 was enriched for ethanol and branched-chain amino acids, particularly L-leucine and L-isoleucine. In contrast, Cluster 1 was markedly enriched for L-arginine.

## Discussion

Developing strains that robustly maintain desired trait expression in industrial applications remains a major challenge in microbial biotechnology. Here, we addressed this challenge with the computational framework TRAEV that can produce predictions on evolutionary robustness of metabolic traits aligned with experimental observations, and thus, complement the existing model-guided strain design methods such as EvolveX [27] for non-GMO strain development or growth-product coupling by genetic engineering [18]. This would alleviate the immense resource need for the proposed experimental robustness screening of strains [10].

Prior to our work, several methods have previously been proposed for predicting and quantifying biological robustness in multiple levels [36–39] (see Section 3 and Table C in S1 Text). In contrast, TRAEV explicitly, and in a novel way, predicts microbial populations’ metabolic trait trajectories of evolutionary adaptation from metabolic trait-fitness couplings inferred from first principles. Thus, the robustness is predicted not only as sensitivity to perturbations but also in terms of how metabolic traits and fitness co-evolve via sub-optimally adapted intermediate states in application environments. The assumption of TRAEV on the adaptation occurring in absence of genetic drift is valid in common ALE experiments (i.e., the transfers of ~1% do not create bottlenecks) including the experimental evolution [34] used to validate TRAEV but importantly also in industrial processes where the population size grows monotonously. TRAEV does not consider clonal interference which is likely to slow down adaptation but not to interfere the monotonous fitness improvement of a population [40,41]. The only potential mechanism for temporarily reduced fitness would be the arise of cheaters. However, their effect on the population fitness is quite rapidly expected to be counteracted by frequency-dependent selection [42]. Thus, monotonous movement toward **v**_a_ in TRAEV can be considered a reasonable approximation of phenotypic evolution of a population. Along the monotonous fitness improvement TRAEV predicts phenotype trajectories that explore alternative solutions. TRAEV’s trajectory-based perspective is particularly relevant for predicting microbial strains’ adaptation in industrial processes, where they undergo notable biomass expansion to production scale and potentially prolonged cultivation.

The two case studies (i.e., pigment and wine aroma production) illustrated proposed applications of TRAEV for designing strategies for robust industrial strain development with biological explainability. In the pigment production case, the relative trait evolutionary robustness in *S. cerevisiae* predicted using TRAEV was consistent with the experimental observations [34], indicating that TRAEV can simulate key adaptive changes in metabolic pathways during a long-term cultivation. At the same time, the explainability of GEM simulations provided a hypothesis of the mechanisms underlying the observed difference in evolutionary robustness. Indigoidine is synthesized from L-glutamine [11], which is directly linked to both the citric acid cycle and nitrogen metabolism, making its synthesis sensitive to many enzyme usage changes in central metabolism when nutrient utilization is already efficient. In contrast, the bikaverin-synthesizing *S. cerevisiae* S288C strain can gain improved fitness via many enzyme usage changes, including those enhancing galactose utilization [34], that do not impair bikaverin synthesis from acetyl-CoA through a relatively insulated pathway [12].

In the aroma production case, TRAEV was used prospectively to evaluate and prioritize selection environments for developing robust non-GMO aroma producing *S. cerevisiae* strains via ALE. The results showed that robust PEA production trait could be developed in *S. cerevisiae* using a narrower spectrum of selection environments, primarily those containing L-phenylalanine, than the BCHA production trait. Both PEA and BCHA aromas are generated via the Ehrlich pathway [43] but L-phenylalanine, the precursor of PEA, is more expensive for yeast to synthesize (e.g., in terms of NADPH requirement) than the branched-chain amino acids [44,45]. In addition to the higher requirement for the supply of reducing power, L-phenylalanine synthesis is more connected to other central pathways (i.e., glycolysis, pentose-phosphate pathway, and nucleotide synthesis) than branched-chain amino acid synthesis. Furthermore, the L-phenylalanine metabolism via Ehrlich pathway involves a specific decarboxylating enzyme (encoded by*ARO10*) while branched-chain amino acid metabolism after deamination occurs by promiscuous enzymes.

Moreover, the nutrient enrichment results were broadly consistent with previous findings. Notably, L-phenylalanine and glycerol were enriched in the cluster of high RS for the PEA production trait, whereas ethanol and L-arginine were enriched in the clusters of high RS for the BCHA production trait. These findings were consistent with the nutrient compositions of the selection environments previously identified by EvolveX for improving the corresponding aroma production [27]. In addition, the association of glycerol-containing environments with more negative TS is consistent with experimental evidence showing that adaptation to glycerol utilization in *S. cerevisiae* can incur fitness costs [46]. Together, these observations suggest that specific nutrient compositions may systematically shape trait robustness and trait-fitness trade-offs during evolutionary adaptation.

Despite the advantages, the current TRAEV implementation is limited by the models it incorporates. First, the modeled application environment did not account for temporal changes during cell growth, such as sequential nutrient depletion or increasing ethanol concentration in a wine fermentation. Modeling time-varying environments (e.g., using dynamic FBA [47]) would allow evolutionary robustness to be assessed under realistic conditions but requires data on compound utilization and production dynamics. Second, incorporating models of trait-fitness dependencies extended beyond metabolism would improve simulation fidelity and predictive accuracy as metabolism is not isolated from other cellular processes.

Ultimately, we envision TRAEV could become a practical component of strain and process development workflows and contribute to enhancing the economic attractiveness of industrial applications using microbial cells.

## Methods

### Models and software

Genome-scale metabolic model (GEM) of *S. cerevisiae* used in this study was Yeast9 GEM (v9.0.2) [35]. The model was transformed using the Gene-Protein-Reaction (GPR) formulation proposed by Machado et al. [32] with the ReFramed package (v1.6.0). Compared with the original GEM, the GPR-transformed model introduces enzyme usage variables and explicitly accounts for complex GPR associations within the metabolic network. MOMA function in ReFramed v1.5.0 was incorporated and used, as it is not available in ReFramed v1.6.0. ReFramed package relies on the COBRApy package (v0.31.1) [48] for metabolic modeling and on the Gurobi Optimizer (v13.0.2) for solving optimization problems.

### Definition of fitness and desired trait

Biomass yield was used as a proxy for the Darwinian fitness *f* of a strain in metabolic state **v**, defined as follows:


$$\begin{array}{c} f(𝐯)=\frac{v_{\mu }}{-\sum_{n\in 𝒩}v_{n}}, \end{array}$$ (1)

where $v_{\mu }$ denotes the flux through the biomass synthesis reaction normalized to produce 1 gDW biomass, and thus represents the specific growth rate (h^–1^). 𝒩 represents the set of carbon and nitrogen source uptake reactions. For each reaction n∈𝒩, $v_{n}$ denotes its flux ($\text{mmol}\cdot {\text{gDW}}^{-1}\cdot {\text{h}}^{-1}$), which is non-positive by the convention of directionality. Thus, the denominator is a positive value representing the total nutrient uptake.

Desired trait *d* of a strain in metabolic state **v** was defined as the yield of the target product(s):


$$\begin{array}{c} d(𝐯)=\frac{\sum_{d\in 𝒟}v_{d}}{-\sum_{n\in 𝒩}v_{n}}, \end{array}$$ (2)

where 𝒟 denotes the set of secretion reactions of the target product(s), and $v_{d}$ represents the flux of each reaction d∈𝒟. The denominator is the same as that in Eq. 1. The reactions included in 𝒟 for all case studies are provided in S1 Data.

For comparison among strains, the desired trait were normalized as follows:


$$\begin{array}{c} d_{norm}(𝐯)=\frac{d(𝐯)}{d_{max}}, \end{array}$$ (3)

where *d*_max_ denotes the normalization baseline. The definition of *d*_max_ depends on the comparison scenario. When comparing different desired traits, *d*_max_ is the desired trait value of the strain in **v**_p_. When comparing the level of the same desired trait across multiple strains, *d*_max_ is instead defined as the maximum desired trait value among all strains in **v**_p_.

### Calculation of v_r_

Metabolic state of the reference strain (**v**_r_) for TRAEV was calculated using parsimonious flux balance analysis (pFBA) [33]. pFBA is an extension of FBA [23] that includes a second optimization step to minimize the total flux after solving the FBA problem, reflecting the principle that cells tend to economize resources.

For simulating adaptive laboratory evolution (ALE), FBA problem was formulated as follows:


$$\begin{array}{cc} \text{minimize}\; & -\sum_{n\in 𝒩}v_{n}\\ \text{subject to}\; & 𝐒𝐯=0,\\ & {𝐯}_{lb}\leq 𝐯\leq {𝐯}_{ub},\\ & v_{\mu }\geq \mu , \end{array}$$ (4)

where **S** is the stoichiometric matrix of the GEM, and **v** represents the metabolic state, i.e., the vector of both metabolic fluxes and enzyme usages. **v**_lb_ and **v**_ub_ denote the lower and upper bounds of **v**, respectively, and the specific growth rate $v_{\mu }$ is constrained to be at least μ.

For simulating genetic modification (GM), the objective in FBA (Eq. 4) was modified to maximization of $\sum_{d\in 𝒟}v_{d}$, and the lower bounds of $v_{n}$ were constrained to physiologically feasible values.

Since using pFBA, after solving Eq. 4 to obtain the optimal objective value (i.e., minimum nutrient uptake), a secondary optimization was performed to minimize the total flux, subject to maintaining the FBA-derived optimal value of the objective function.

### TRAEV

We proposed the TRAEV framework to perform Simulation and Robustness Analysis of adaptive evolution of developed strains in application environments. In Simulation, we calculated the strain’s immediate phenotypically adjusted metabolic state (**v**_p_) and evolutionarily adapted metabolic state (**v**_a_) in an application environment.

#### Calculation of v_p_.

Calculation of **v**_p_ was implemented using linear MOMA [30], a linear version of classical (quadratic) MOMA [24], with **v**_r_ as the reference state. The objective of quadratic MOMA is to minimize the Euclidean (L2-norm) distance to the wild-type (reference) state, denoted as **w**, and the problem is formulated as follows:


$$\begin{array}{cc} \text{minimize}\; & \sum_{i\in 𝒰}(v_{i}-w_{i}{)}^{2}\\ \text{subject to}\; & 𝐒𝐯=0,\\ & v_{lb}\leq 𝐯\leq {𝐯}_{ub},\\ & v_{\mu }\geq \mu ,\\ & i\in 𝒰, \end{array}$$ (5)

where **S** is the stoichiometric matrix of the GEM, and **v** represents the metabolic state, i.e., the vector (distribution) of both metabolic fluxes and enzyme usages. **v**_lb_ and **v**_ub_ denote the lower and upper bounds of **v**, respectively, and the growth rate $v_{\mu }$ was constrained to be at least μ. In this study, μ was set to 0.005 ~ h^–1^, a biologically plausible minimum growth rate. $v_{i}$ denotes the *i*-th component of **v** and $w_{i}$ denotes the *i*-th component of **w**. 𝒰 denotes the set of components appearing in the objective function. Since the GPR-transformed model [32] was used in this study, 𝒰 corresponds to all enzyme usage variables.

MOMA assumes that following a sudden perturbation (e.g., an environmental shift), cells transiently adopt a metabolic state that minimizes deviation from the original (reference) state rather than immediately reaching a new optimal state [24]. It captures the continuous and distributed nature of short-term metabolic reorganization, in which many fluxes undergo small yet coordinated adjustments constrained by the reference state. Such behavior is consistent with phenotypic plasticity, which involves immediate, non-genetic physiological adjustments to environmental perturbations and often precedes long-term evolutionary adaptation [28,29]. Therefore, MOMA provides a biologically grounded framework for simulating short-term phenotypic plastic responses following environmental perturbations.

In linear MOMA, the objective function in Eq. 5 is replaced with the L1-norm distance $\sum_{i\in 𝒰}|\;v_{i}-w_{i}|$. Quadratic MOMA penalizes large deviations more strongly and tends to distribute flux changes across many reactions. In contrast, linear MOMA allows larger deviations in a smaller subset of reactions, which can lead to more localized flux rerouting and potentially improved biological interpretability [30,49].

#### Calculation of v_a_.

Calculation **v**_a_ was implemented using an iterative search algorithm (Algorithm 1) using ROOM [31]. The objective of ROOM is to minimize the total number of flux and/or enzyme usage changes relative to the wild-type (reference) state, denoted as **w**, and the problem is formulated as follows:


$$\begin{array}{cc} \text{minimize}\; & \sum_{i\in 𝒰}y_{i}\\ \text{subject to}\; & 𝐒𝐯=0,\\ & {𝐯}_{lb}\leq 𝐯\leq {𝐯}_{ub},\\ & v_{\mu }\geq \mu ,\\ & v_{i}-y_{i}(v_{lb,i}-w_{lb,i})\geq w_{lb,i},\\ & v_{i}-y_{i}(v_{ub,i}-w_{ub,i})\leq w_{ub,i},\\ & w_{lb,i}=w_{i}-\delta |w_{i}|-\varepsilon ,\\ & w_{ub,i}=w_{i}+\delta |w_{i}|+\varepsilon ,\\ & y_{i}\in \{0,1\},\\ & i\in 𝒰, \end{array}$$ (6)

where **S** is the stoichiometric matrix of the GEM, and **v** represents the metabolic state, i.e., the vector (distribution) of both metabolic fluxes and enzyme usages. **v**_lb_ and **v**_ub_ denote the lower and upper bounds of **v**, respectively, and the specific growth rate $v_{\mu }$ is constrained to be at least μ. $y_{i}$ is a binary variable representing whether the value of $v_{i}$ (*i*-th component of **v**) falls outside the wild-type range [*w*_lb,*i*_, *w*_ub,*i*_]. The bounds *w*_ub, *i*_ and *w*_lb, *i*_ are derived from the corresponding $w_{i}$ (*i*-th component of **w**) using the relative tolerance δ and the absolute tolerance ε. In this study, δ=0.01 and ε=0.0001 were used to define a relatively narrow tolerance range, enabling small deviations of $v_{i}$ from $w_{i}$ to be classified as changes, i.e., $y_{i}=1$. 𝒰 denotes the set of components appearing in the objective function. In this study, 𝒰 corresponds to all enzyme usage variables.

ROOM assumes that following a genetic perturbation (e.g., a gene loss of function), cells reorganize their metabolism by minimizing the number of significant flux changes relative to the original (reference) state, rather than minimizing the overall magnitude of flux deviations [31]. In contrast to the continuous and distributed flux adjustments captured by MOMA, ROOM favors sparse metabolic reorganization in which only a limited subset of reactions undergo substantial changes while the majority remain largely unaffected. This behavior is consistent with responses to limited number of genetic mutations, during which a stable metabolic state is progressively established under new genotype [50,51]. Therefore, ROOM provides a biologically grounded framework for simulating metabolic reorganization following genetic perturbations allowing adaptive evolution.

To identify the evolutionarily adapted state **v**_a_, we employed the iterative search algorithm described in Algorithm 1. The phenotypically adjusted state was used as the reference state (i.e., **w** = **v**_p_), with the corresponding growth rate $\mu_{p}$ serving as the initial value of μ. The algorithm then incrementally increased μ by a fixed step size Δμ and computed the corresponding ROOM solution at each step. The search terminated when the fitness predicted by ROOM reached a near-optimal threshold *f*_opt_, defined as 95% of the fitness predicted by FBA (Eq. 4) under the same constraints, or when μ reached the upper bound $\mu_{max}$. Here, $\mu_{max}$ denotes the maximum biologically plausible growth rate achievable by the target species or strain in the application environment and was estimated from literature reports, experimental measurements, or model-based approximations. During the search, the μ value associated with the highest ROOM-predicted fitness encountered was recorded as $\mu_{best}$, and the corresponding ROOM solution was taken as **v**_a_. A step size of $\Delta \mu =0.05\;h^{-1}$ was used as a compromise between computational efficiency and search resolution. After identifying a coarse-grained estimate of $\mu_{best}$, a fine-grained search with $\Delta \mu =0.005\;h^{-1}$ was performed around this estimate to improve precision.

**Algorithm 1 Iterative search algorithm for v_a_**

**Require:** GEM **S**;

reference state **v**_p_ and growth rate $\mu_{p}$;

maximum growth rate $\mu_{max}$;

optimal fitness *f*_opt_;

growth rate increment Δμ

**Ensure:** adapted state **v**_a_

1: Initialize growth rate $\mu \leftarrow \mu_{p}$ and $\mu_{best}\leftarrow \mu$

2: Initialize fitness f←−∞ and $f_{best}\leftarrow f$

3: **while** $\mu \leq \mu_{max}$ **do**

4:  $f\leftarrow f{|}_{\text{ROOM}(𝐒,{𝐯}_{p},\mu )}$

5:  **if** *f* > *f*_best_ **then**

6:   $f_{best}\leftarrow f$

7:   $\mu_{best}\leftarrow \mu$

8:  **end if**

9:  **if** $f\geq f_{opt}$ **then**

10:   **break**

11:  **end if**

12:  μ←μ+Δμ

13: **end while**

14: **return** ${𝐯}_{a}\leftarrow 𝐯{|}_{\text{ROOM}(𝐒,{𝐯}_{p},\mu_{best})}$

#### Robustness analysis.

Following the Simulation, the Robustness Analysis was performed through a Monte Carlo-based perturbation analysis method. Since the specific genetic mutations that occurred during evolution are unknown, we considered the changes in enzyme usage observed between **v**_p_ and **v**_a_ as phenotypic proxies of the effects of underlying genetic perturbations. Therefore, each enzyme usage change was regarded as a perturbation event in this analysis.

Let ℳ denote the set of perturbations (i.e., changes in enzyme usage) between **v**_p_ and **v**_a_, and let |ℳ| be its cardinality. For each enzyme usage variable *i*, the corresponding binary variable $y_{i}$ in Eq. 6 was considered to represent an enzyme usage change when $y_{i}\geq 0.5$. For each perturbation count k∈{1,2,...,|ℳ|}, there are (|ℳ|k) possible perturbation combinations. From these (|ℳ|k) perturbation combinations, we randomly selected a sample *s*, which is a subset of ℳ and its corresponding evolutionarily intermediate metabolic state was defined as follows:


$$\begin{array}{c} {𝐯}_{s}={𝐯}_{p}+{𝐦}_{s}⊙({𝐯}_{a}-{𝐯}_{p}) \end{array}$$ (7)

where ${𝐦}_{s}$ is a binary mask whose component is 1 if the corresponding perturbation is included in *s* and 0 otherwise. To guarantee the feasibility of ${𝐯}_{s}$, we did not use Eq. 7 directly. Instead, quadratic MOMA (Eq. 5) was run with **v**_p_ as the reference state and an additional constraint that the perturbations in *s* were fixed to their values in **v**_a_. If MOMA returned a feasible solution, it was taken as the feasible approximation of ${𝐯}_{s}$, where the components in *s* equal to the values in **v**_a_, whereas the remaining components are kept close to their values in **v**_p_.

We repeated the above sampling procedure to generate up to *n* evolutionary trajectories. Starting from a randomly selected perturbation combination with *k* = 1, one additional perturbation was successively added until *k* = *m*, and for each perturbation combination the corresponding feasible evolutionarily intermediate metabolic state was obtained using the procedure described above. Thus, each trajectory contained up to *m* evolutionarily intermediate metabolic states between **v**_p_ and **v**_a_. Collectively, the *n* trajectories yielded a set of states $𝒱=\left\{{𝐯}_{s}|s\in 𝒮\right\}$, where 𝒮 denotes the set of sampled perturbation combinations with |𝒮|≤m×n. Here, *m* and *n* are predefined sampling parameters denoting the maximum perturbation count and the number of sampled trajectories, respectively, with m≤|ℳ|.

Next, the fitness (Eq. 1) and the desired trait (Eq. 2) values were derived from 𝒱. The desired trait values were then normalized using Eq. 3. The average evolutionary trajectory was then obtained from the mean normalized fitness and desired trait values across all states ${𝐯}_{s}\in 𝒱$ satisfying |*s*| = *k*. In addition, the cumulative trait-fitness trade-off up to *k* was quantified by the Pearson correlation coefficient between $\Delta d({𝐯}_{s})$ and $\Delta f({𝐯}_{s})$ [$\Delta d({𝐯}_{s})=d({𝐯}_{s})-d({𝐯}_{p})$ and $\Delta f({𝐯}_{s})=f({𝐯}_{s})-f({𝐯}_{p})$] using all states ${𝐯}_{s}\in 𝒱$ satisfying |s|≤k.

Finally, the Robustness Score (RS) and Trade-off Score (TS) based on 𝒱 were calculated as follows:


$$\begin{array}{c} RS=\frac{1}{\left|𝒱\right|}\sum_{{𝐯}_{s}\in 𝒱}d_{norm}({𝐯}_{s}), \end{array}$$ (8)

$$\begin{array}{c} TS=corr\left({\left\{\Delta d({𝐯}_{s})\right\}}_{{𝐯}_{s}\in 𝒱},{\left\{\Delta f({𝐯}_{s})\right\}}_{{𝐯}_{s}\in 𝒱}\right). \end{array}$$ (9)

The range of RS is [0,1], where larger values indicate higher trait robustness and/or attainable trait level. The range of TS is [−1,1], corresponding to the range of the Pearson correlation coefficient. A non-negative TS (TS≥0) indicates the absence of a trait-fitness trade-off, whereas a negative TS (TS < 0) indicates the presence of a trade-off. More negative values correspond to stronger trade-offs between the desired trait and fitness.

### Experimental procedures

#### Simulations for predicting the evolutionary robustness of heterologous pigment production in *S. cerevisiae.*

We augmented the yeast consensus GEM (v9.0.2) [35] for two engineered *S. cerevisiae* strains, the indigoidine-producing CEN.PK113-7D and the bikaverin-producing S288C, by incorporating the corresponding heterologous reactions (see Sections 1.1 and 1.2 in S1 Text). When the heterologous pathways included native metabolites of yeast, the native metabolite identifiers in the GEM were used for creating the appropriate connectivity of the heterologous pathways to the native metabolism. The secretion flux of the corresponding pigment was defined as the desired trait (see S1 Data).

For each strain, the engineering environment was set under aerobic conditions with D-glucose as the sole carbon source and ammonium as the sole nitrogen source. The growth rate (μ) was set to 0.2 ~ h^–1^, and the D-glucose uptake flux was capped at $10\;mmol\cdot {gDW}^{-1}\cdot h^{-1}$, while ammonium uptake flux was unconstrained ($\leq 1000\;mmol\cdot {gDW}^{-1}\cdot h^{-1}$) to mimic D-glucose limited growth. pFBA was then performed to maximize the corresponding pigment secretion flux, and the resulting metabolic state was used as the reference state (**v**_r_) for TRAEV.

Next, we applied TRAEV to each strain in the two application environments: YNBG (ammonium + D-galactose) and YPG (peptone + D-galactose). For the Simulation in YNBG, the D-galactose uptake flux was unconstrained, whereas the ammonium uptake flux was capped at $10\text{ mmol}\cdot {\text{gDW}}^{-1}\cdot {\text{h}}^{-1}$; for the Simulation in YPG, the D-galactose uptake flux was unconstrained, whereas uptake flux of each of the 20 standard amino acids was capped at $1\text{ mmol}\cdot {\text{gDW}}^{-1}\cdot {\text{h}}^{-1}$. The maximum growth rates ($\mu_{max}$) were set to 0.4 ~ h^–1^ for YNBG and 0.6 ~ h^–1^ for YPG, according to [52]. Using the metabolic states **v**_p_ and **v**_a_ resulting from the Simulation and the sampling parameters *m* = 50 and *n* = 100, the Robustness Analysis was performed to obtain the evolutionary trajectories, RS and TS. We choose *m* = 50 because the total numbers of enzyme usage changes between **v**_p_ and **v**_a_ were between 60 and 80.

#### Simulations for predicting the evolutionary robustness of aroma production in evolved *S. cerevisiae* strains under different selection environments.

We adjusted the yeast consensus GEM (v9.0.2) [35] to favor aroma compound production (see Section 1.3 in S1 Text). The desired traits for PEA and BCHA production were defined as the sum of fluxes through the corresponding aroma secretion reactions (listed in S1 Data).

To simulate the aerobic ALE process in each selection environment, the uptake fluxes of nutrients present in the environment were set to unconstrained ($\leq 1000\text{mmol}\cdot {\text{gDW}}^{-1}\cdot {\text{h}}^{-1}$), while the uptake fluxes of nutrients absent from the environment were set to zero. The growth rate $\mu =0.4\;h^{-1}$ was enforced. pFBA was then performed with the objective of minimizing total nutrient uptake. Simulations were conducted across all 1171 selection environments (see S2 Data), resulting in 1171 reference metabolic states (**v**_p_).

For each reference state, we applied TRAEV to calculate the metabolic states **v**_p_ and **v**_a_ in the anaerobic wine must-like environment (see Section 1.4 in S1 Text). Next, using the Simulation results and the sampling parameters *m* = 50 and *n* = 100, the Robustness Analysis was performed to obtain the evolutionary trajectories, RS and TS. We choose *m* = 50 because the total numbers of enzyme usage changes between **v**_p_ and **v**_a_ were typically between 50 and 100.

We ran the program across all 1,171 selection environments in parallel on the Aalto Triton cluster with 40 CPU cores, requiring approximately one day of wall-clock time.

### Selection environment clustering and nutrient enrichment analysis

Only selection environments that yielded evolved strains capable of producing the target aroma in the application environment, and therefore generated valid Robustness Analysis results, were included in this analysis. This criterion retained 322 of the 1,171 selection environments for the PEA production trait and 499 of the 1,171 selection environments for the BCHA production trait.

For each aroma production trait, each selection environment was represented by its corresponding RS and TS. These scores were standardized and clustered into four groups using K-means clustering implemented in the sklearn Python package. For each cluster, the number of environments and the mean and standard deviation of RS and TS were calculated.

Nutrient enrichment analysis was then conducted to identify features overrepresented in each cluster. The features for this analysis were defined as either single nutrients or nutrient pairs derived from the nutrient compositions of the selection environments. The nutrient pairs in which both components were carbon-source-only substrates, such as D-glucose and D-galactose, were excluded from the analysis. For each feature *j* in cluster *c*, fold enrichment was quantified as:


$$\begin{array}{c} {FE}_{cj}=\frac{n_{cj}/N_{c}}{n_{j}/N}, \end{array}$$ (10)

where $n_{cj}$ is the number of environments in cluster *c* containing feature *j*, $N_{c}$ is the total number of environments in cluster *c*, $n_{j}$ is the number of environments containing feature *j* across all environments, and *N* is the total number of environments.

To reduce the influence of features with high fold enrichment but limited occurrence within a cluster, an enrichment score was calculated as:


$$\begin{array}{c} {ES}_{cj}=\log_{2}({FE}_{cj})\sqrt{n_{cj}}. \end{array}$$ (11)

This score combines the fold enrichment of a feature with the number of environments containing that feature within the cluster.

For each feature *j*, the mean RS and TS across the environments containing that feature within the corresponding cluster were also computed to characterize the associated RS/TS profile.

### Sensitivity analysis

Sensitivity analysis was conducted to evaluate whether the RS and TS rankings of the compared groups depended on the parameter settings in TRAEV. For both the pigment and aromatic production case studies, the TRAEV Robustness Analysis was rerun using nine combinations of the maximum perturbation count m∈{10,20,50} and the number of sampled trajectories n∈{30,50,100}, with the relative tolerance δ=0.01 and the absolute tolerance ε=0.0001. Additionally, two alternative tolerance settings, (δ,ε)=(0.03,0.001) and (0.1, 0.01) were tested with (*m*, *n*) = (50, 100).

For the pigment production case study, RS and TS were recalculated for the two pigments in each medium under all parameter settings. For the aromatic production case study, the four TS/RS clusters of the environments for each aroma were identified under the (m,n,δ,ε)=(50,100,0.01,0.0001) parameter setting. RS and TS were recalculated for the four RS/TS clusters under all parameter settings. Parameter sensitivity was assessed by determining whether the relative rankings of pigments and aromatic RS/TS clusters were preserved across all parameter settings.

## Supporting information

S1 Text Supplementary information. Additional information on strain and environment modeling, results of sensitivity analysis, comparison with previous methods, and summary of symbols and abbreviations.
(PDF)

S1 Fig Pigment production sensitivity analysis. RS and TS were recalculated using eleven parameter settings. (A, B) RS under different parameter settings in YNBG (A) and YPG (B) media, respectively. (C, D) TS under different parameter settings in YNBG (C) and YPG (D) media, respectively.
(TIF)

S2 Fig Aromatic production sensitivity analysis. RS and TS were recalculated using eleven parameter settings. (A, B) RS under different parameter settings for the PEA (A) and BCHA (B) production traits, respectively. (C, D) TS under different parameter settings for the PEA (C) and BCHA (D) production traits, respectively.
(TIF)

S1 Data Desired traits. List of metabolic reactions representing the desired trait of strains.
(XLSX)

S2 Data Selection environments. List of selection environments for ALE of aroma production strains.
(XLSX)
