# Supplementary material for: In silico prediction of metabolic trait robustness in microbial cells
Source: PLoS Comput Biol. 2026 Oct 27;22(10):e1014864. doi: 10.1371/journal.pcbi.1014864 (PMC13727050; doi:10.1371/journal.pcbi.1014864)
Supplement: Supplementary file 1 [file pcbi.1014864.s001.pdf]

In silico prediction of metabolic trait robustness in microbial cells

Chunhao Gu[1], Ville Mustonen[2], Paula Jouhten[1*]

1 Department of Bioproducts and Biosystems, Aalto University, Espoo, Finland
2 Department of Computer Science, Department of Organismal and Evolutionary Biology, University of Helsinki, Helsinki, Finland

* paula.jouhten@aalto.fi

1 GEM modifications

The wild-type genome-scale metabolic model (GEM) used in this study was the yeast consensus model (v9.0.2, `https://zenodo.org/records/14210050`) [1]. In addition, the reactions (2R,3S)-3-methylmalate:NAD$^+$ oxidoreductase (`r_4581`) and malate/β-methylmalate synthase (`r_4582`) were closed. Both reactions are annotated with Confidence Level 0 and lack gene associations; therefore, closing them can avoid spurious side routes and keep flux in well-supported metabolic pathways.

1.1 Indigoidine-producing strain

The GEM for an indigoidine-producing strain was constructed by incorporating the indigoidine biosynthetic pathway into the yeast consensus model. The pathway comprises a heterologous gene encoding indigoidine synthetase (BpsA) and a phosphopantetheinyl transferase (PPTase). BpsA is a nonribosomal peptide synthetase (NRPS) that catalyzes the condensation of two L-glutamine molecules to form one indigoidine molecule, while PPTase activates BpsA by converting it from its apo- to holo-form [2]. Because the PPTase reaction is a post-translational modification that does not influence metabolic fluxes, it was excluded from the model. Accordingly, the indigoidine biosynthetic pathway was represented as a single overall reaction based on the stoichiometry described in [3], omitting the FMN-dependent step since the flavin cofactor functions as an enzyme-bound prosthetic group and therefore does not carry metabolic flux. The added synthesis, transport, and exchange reactions are listed in Table A.

Table A. Reactions added for the indigoidine biosynthetic pathway.

No.	Reaction Name	Stoichiometry	Compartment	Gene Association
1	Indigoidine synthase	2L-glutamine + 2ATP + O_2 $\rightarrow$ Indigoidine[c] + 2AMP + 2PP_i + 2H_2O	cytosol	*bpsA*
2	Indigoidine transport	Indigoidine[c] $\rightleftharpoons$ Indigoidine[e]	cytosol $\rightleftharpoons$ extracellular	–
3	Indigoidine exchange	Indigoidine[e] $\rightarrow$	extracellular	–

1.2 Bikaverin-producing strain

The GEM for an bikaverin-producing strain was constructed by incorporating the bikaverin biosynthetic pathway into the yeast consensus model. The pathway essentially comprises three heterologous genes encoding one polyketide synthase (Bik1) and two tailoring enzymes (Bik2, Bik3), and a phosphopantetheinyl transferase (PPTase). Bik1, activated by PPTase, condenses one acetyl-CoA and eight malonyl-CoA units to form pre-bikaverin, which is subsequently oxidized and methylated by Bik2 (monooxygenase) and Bik3 (O-methyltransferase) to produce bikaverin [4]. Thus, the bikaverin biosynthesis was represented as a single overall reaction catalyzed by Bik1-3. The added synthesis, transport, and exchange reactions are listed in Table B.

Table B. Reactions added for the bikaverin biosynthetic pathway.

No.	Reaction name	Stoichiometry	Compartment	Gene association
1	Bikaverin synthase	8Malonyl-CoA + Acetyl-CoA + 2SAM + $2O_2$ + 2NADPH$\rightarrow$ Bikaverin[c] + 9CoA + $8CO_2$ + 2SAH + $2H_2O$ + $2NADP^+$	cytosol	*bik1*, *bik2*, and *bik3*
2	Bikaverin transport	Bikaveri[c] $\rightleftharpoons$ Bikaverin[e]	cytosol $\rightleftharpoons$ extracellular	–
3	Bikaverin exchange	Bikaverin[e] $\rightarrow$	extracellular	–

1.3 Aroma-producing strain

For the aroma-producing strains, both the phenylethyl alcohol (PEA) and branched-chain higher alcohol (BCHA) pathways were adjusted in the GEM to favor aroma compound synthesis while remaining physiologically reasonable. Specifically, the reactions isoamyl acetate-hydrolyzing esterase (`r_0656`) and isobutyl acetate-hydrolyzing esterase (`r_0657`) were closed. These constraints suppress the hydrolysis of higher alcohol acetate esters, eliminate futile ester cycles, and enable the model to reproduce the increased accumulation of acetate esters.

Additionally, the phenylacetaldehyde secretion (`r_2001`) was closed. Phenylacetaldehyde is an intermediate of the Ehrlich pathway that is typically reduced intracellularly to PEA rather than secreted. Closing its secretion in the GEM redirects the flux toward PEA formation.

1.4 Wine must environment

The wine must environment represents the nutrient and physicochemical conditions that yeast *Saccharomyces cerevisiae* encounters during grape must fermentation. This environment is largely (after early phase) anaerobic, with D-glucose and D-fructose serving as the primary carbon sources and yeast assimilable nitrogen (ammonium and free amino acids) as the main nitrogen sources. Under these conditions, yeast metabolism relies predominantly on fermentation to produce ethanol, glycerol, and aroma compounds, while oxidative respiration remains inactive.

To simulate this environment *in silico*, the GEM was configured to allow unconstrained uptake ($\leq 1000 \mathrm{mmol} \cdot \mathrm{gDW}^{-1} \cdot \mathrm{h}^{-1}$) of D-glucose (`r_1714`), D-fructose (`r_1709`), and ammonium (`r_1654`), while capping uptake of each of the 20 standard amino acids (`r_1873`, `r_1879`, `r_1880`, `r_1881`, `r_1883`, `r_1889`, `r_1891`, `r_1810`, `r_1893`, `r_1897`, `r_1899`, `r_1900`, `r_1902`, `r_1903`, `r_1904`, `r_1906`, `r_1911`, `r_1912`, `r_1913`, `r_1914`) at $1\ \mathrm{mmol} \cdot \mathrm{gDW}^{-1} \cdot \mathrm{h}^{-1}$ to prevent them from serving as major carbon sources. Anaerobiosis was mimicked by closing reactions associated with oxidative

respiration, including cytochrome c oxidase (`r_0438`), succinate dehydrogenase (`r_1021`), mitochondrial ATP synthase (`r_0226`), and FMN reductase (`r_0441`, `r_0441`).

Furthermore, reactions involved in glycerol catabolism (`r_0487`, `r_0488`) and glyoxylate cycle activity (`r_0662`) were closed to ensure that intracellularly produced glycerol serves primarily to maintain redox balance rather than be fed back into central carbon metabolism. This is consistent with experimental evidence that glycerol accumulates and is secreted during anaerobic fermentation.

In addition, isocitrate dehydrogenase (`r_0659`), glycine hydroxymethyltransferase (`r_0502`, `r_0503`), and methylenetetrahydrofolate dehydrogenase (`r_0732`, `r_0733`), were constrained to proceed only in their physiologically forward directions. Because the reverse directions of these reactions are thermodynamically unfavorable under fermentative conditions, applying directional constraints ensures physiologically realistic NADH/NADPH cofactor balances.

2 Results of sensitivity analysis

To evaluate the sensitivity of TRAEV results to parameter settings, the Robustness Scores (RS) and Trade-off Scores (TS) were recalculated across nine combinations of the maximum perturbation count $m \in \{10, 20, 50\}$ and the number of sampled trajectories $n \in \{30, 50, 100\}$, with the relative tolerance $\delta = 0.01$ and the absolute tolerance $\varepsilon = 0.0001$. Additionally, two alternative tolerance settings, $(\delta, \varepsilon) = (0.1, 0.01)$ and $(0.03, 0.001)$, were tested with $(m, n) = (50, 100)$.

For the pigment production case study, the relative RS and TS rankings of the two pigment-producing strains were preserved across all eleven parameter settings (S1 Fig). In both YNBG and YPG media, the bikaverin-producing strain consistently exhibited higher RS values than the indigoidine-producing strain (S1A and S1B Fig). Likewise, the bikaverin-producing strain maintained higher TS values than the indigoidine-producing strain under all parameter settings (S1C and S1D Fig). Although the absolute RS and TS values varied with the parameter settings, the relative RS and TS rankings remained unchanged.

For the aromatic production case study, the relative RS and TS rankings were assessed for the four clusters identified under $(m, n, \delta, \varepsilon) = (50, 100, 0.01, 0.0001)$ (shown in Fig 4 of the main manuscript). Although the absolute scores varied, the overall ordering patterns were preserved across the nine combinations of m and n with $(\delta, \varepsilon) = (0.01, 0.0001)$ (S2 Fig). For the PEA production trait, the overall RS ordering remained

$$\mathrm{RS}_3 > \mathrm{RS}_1 > \mathrm{RS}_0 \approx \mathrm{RS}_2$$

(S2A Fig), while the overall TS ordering was

$$\mathrm{TS}_0 \approx \mathrm{TS}_1 > \mathrm{TS}_2 > \mathrm{TS}_3$$

(S2C Fig). For the BCHA production trait, the overall RS ranking was maintained as

$$\mathrm{RS}_3 > \mathrm{RS}_1 > \mathrm{RS}_2 > \mathrm{RS}_0$$

(S2B Fig), and the overall TS ranking remained

$$\mathrm{TS}_1 > \mathrm{TS}_2 > \mathrm{TS}_3 \approx \mathrm{TS}_0$$

(S2D Fig).

The two additional tolerance settings, $(\delta, \varepsilon) = (0.03, 0.001)$ and $(0.1, 0.01)$, showed the same overall RS ordering patterns as $(\delta, \varepsilon) = (0.01, 0.0001)$ for both PEA and BCHA. However, TS differences between some clusters narrowed, and some relative rankings changed. For PEA, TS_0 and TS_1 became closer to TS_2, while TS_3 consistently remained the lowest. For BCHA, TS_1 and TS_2 remained higher than TS_0 and TS_3, but TS_2 exceeded TS_1 under both additional tolerance settings. The gaps among TS_0, TS_1, and TS_2 also narrowed, particularly at $(\delta, \varepsilon) = (0.1, 0.01)$.

Overall, these results supported the stability of RS and TS rankings for the pigment production case study. For the aromatic production case study, the overall RS and TS ordering patterns were largely preserved across the tested parameter settings, although some TS rankings changed with the tolerance settings. The alternative tolerance settings defined wider tolerance ranges, resulting in the detection of fewer enzyme usage changes, predominantly those of larger magnitude, than under the default tolerance setting, $(\delta, \varepsilon) = (0.01, 0.0001)$. This difference affected the Robustness Analysis and TS results to some extent.

3 Comparison of TRAEV with previous studies

Previous studies have investigated robustness at different biological levels and using distinct methodological frameworks. Kitano's theory [5] provides a conceptual framework for biological robustness, defining it in terms of the maintenance of biological functions under perturbations. At the metabolic network level, Yang et al. [6] quantified robustness using the variation in target metabolite production across predefined perturbations, whereas Gerstl et al. [7] estimated robustness as the probability of failure of a specified network function based on minimal cut sets (MCSs). Trivellin et al. [8] developed a data-driven framework to quantify the robustness of experimental phenotypes across perturbation conditions using the Fano factor.

In contrast, EvolveX [9] uses genome-scale metabolic models (GEMs) to evaluate the evolvability of metabolic traits by identifying evolution environments that can promote a desired trait in an application environment. TRAEV extends this perspective by jointly evaluating robustness and evolvability at the level of metabolic traits using GEMs. Specifically, TRAEV quantifies the robustness of a desired trait along predicted evolutionary trajectories and characterizes the trait-fitness trade-off during adaptation to the application environment. Thus, TRAEV enables a trajectory-based assessment of whether a desired metabolic trait can be maintained during evolutionary adaptation.

Table C compares TRAEV with EvolveX and representative previous studies on biological robustness.

Table C. Comparison of TRAEV with EvolveX and representative previous studies on biological robustness.

Study	Level	Approach	Robustness	Evolvability
Kitano [5]	Biological systems	Conceptual	✓	–
Yang et al. [6]	Metabolic networks	GEM-based	✓	–
Gerstl et al. [7]	Metabolic networks	GEM-based	✓	–
Trivellin et al. [8]	Experimental phenotypes	Data-driven	✓	–
EvolveX [9]	Metabolic traits	GEM-based	–	✓
TRAEV	Metabolic traits	GEM-based	✓	✓

Note: A check mark indicates that the corresponding property is explicitly quantified as a primary output of the framework, while an en dash indicates that it is not explicitly quantified.

4 Summary of TRAEV symbols and abbreviations

Table D summarizes the key symbols and abbreviations used to describe the TRAEV framework in the main manuscript.

Table D. Summary of key symbols and abbreviations used to describe TRAEV.

Symbol/ Abbr.	Name	Description
$\mathbf{v}_\mathrm{r}$	Reference metabolic state	Input to TRAEV Simulation. The metabolic state of a developed strain (e.g., an engineered or laboratory-evolved strain). This state can be calculated using parsimonious flux balance analysis (pFBA) [10].
$\mathbf{v}_\mathrm{p}$	Phenotypically adjusted metabolic state	Calculated by TRAEV Simulation. The immediate metabolic state due to phenotypic plasticity after switching to the application environment, calculated using linear minimization of metabolic adjustment (linear MOMA) [11, 12] similarly to [13]. This state minimizes the distance from $\mathbf{v}_\mathrm{r}$ while ensuring a biologically viable minimal growth rate.
$\mathbf{v}_\mathrm{a}$	Evolutionarily adapted metabolic state	Calculated by TRAEV Simulation. The adapted metabolic state after evolution in the application environment, calculated using the regulatory on/off minimization (ROOM) [14]-based iterative search algorithm (Algorithm 1 in the main manuscript). This state achieves optimal fitness with the fewest changes in enzyme usage (i.e., phenotypic effects of mutations) relative to $\mathbf{v}_\mathrm{p}$.
RS	Robustness Score	Calculated by TRAEV Robustness Analysis. A metric defined as the mean, over evolutionarily intermediate metabolic states, of the normalized desired trait, reflecting the evolutionary robustness and overall level of the desired trait.
TS	Trade-off Score	Calculated by TRAEV Robustness Analysis. A metric defined as the Pearson correlation coefficient, over evolutionarily intermediate metabolic states, between the changes (relative to $\mathbf{v}_\mathrm{p}$) in the desired trait and in fitness, representing the trait-fitness trade-off (i.e., negative coupling).

References

1. Zhang C, Sánchez BJ, Li F, Eiden CWQ, Scott WT, Liebal UW, et al. Yeast9: A Consensus Genome-Scale Metabolic Model for S. Cerevisiae Curated by the Community. Molecular Systems Biology. 2024;20(10):1134–1150. doi:10.1038/s44320-024-00060-7.

2. Wehrs M, Prahl JP, Moon J, Li Y, Tanjore D, Keasling JD, et al. Production Efficiency of the Bacterial Non-Ribosomal Peptide Indigoidine Relies on the Respiratory Metabolic State in S. Cerevisiae. Microbial Cell Factories. 2018;17(1):193. doi:10.1186/s12934-018-1045-1.

3. EC 4.3.3.9;. https://iubmb.qmul.ac.uk/enzyme/EC4/3/3/9.html.

4. Zhao M, Zhao Y, Yao M, Iqbal H, Hu Q, Liu H, et al. Pathway Engineering in Yeast for Synthesizing the Complex Polyketide Bikaverin. Nature Communications. 2020;11(1):6197. doi:10.1038/s41467-020-19984-3.

5. Kitano H. Towards a Theory of Biological Robustness. Molecular Systems Biology. 2007;3(1):137. doi:10.1038/msb4100179.

6. Yang L, Srinivasan S, Mahadevan R, Cluett WR. Characterizing Metabolic Pathway Diversification in the Context of Perturbation Size. Metabolic Engineering. 2015;28:114–122. doi:10.1016/j.ymben.2014.11.013.

7. Gerstl MP, Klamt S, Jungreuthmayer C, Zanghellini J. Exact Quantification of Cellular Robustness in Genome-Scale Metabolic Networks. Bioinformatics. 2016;32(5):730–737. doi:10.1093/bioinformatics/btv649.

8. Trivellin C, Olsson L, Rugbjerg P. Quantification of Microbial Robustness in Yeast. ACS Synthetic Biology. 2022;11(4):1686–1691. doi:10.1021/acssynbio.1c00615.

9. Jouhten P, Konstantinidis D, Pereira F, Andrejev S, Grkovska K, Castillo S, et al. Predictive Evolution of Metabolic Phenotypes Using Model-designed Environments. Molecular Systems Biology. 2022;18(10):e10980. doi:10.15252/msb.202210980.

10. Lewis NE, Hixson KK, Conrad TM, Lerman JA, Charusanti P, Polpitiya AD, et al. Omic Data from Evolved E. Coli Are Consistent with Computed Optimal Growth from Genome-scale Models. Molecular Systems Biology. 2010;6(1):390. doi:10.1038/msb.2010.47.

11. Segrè D, Vitkup D, Church GM. Analysis of Optimality in Natural and Perturbed Metabolic Networks. Proceedings of the National Academy of Sciences. 2002;99(23):15112–15117. doi:10.1073/pnas.232349399.

12. Becker SA, Feist AM, Mo ML, Hannum G, Palsson BØ, Herrgard MJ. Quantitative Prediction of Cellular Metabolism with Constraint-Based Models: The COBRA Toolbox. Nature Protocols. 2007;2(3):727–738. doi:10.1038/nprot.2007.99.

13. Ho WC, Zhang J. Evolutionary Adaptations to New Environments Generally Reverse Plastic Phenotypic Changes. Nature Communications. 2018;9(1):350. doi:10.1038/s41467-017-02724-5.

14. Shlomi T, Berkman O, Ruppin E. Regulatory on/off Minimization of Metabolic Flux Changes after Genetic Perturbations. Proceedings of the National Academy of Sciences. 2005;102(21):7695–7700. doi:10.1073/pnas.0406346102.
